# Genomic packaging and epigenetic regulation of genes

**DOI:** 10.1186/1755-8166-7-S1-I25

**Published:** 2014-01-21

**Authors:** Rakesh Mishra

**Affiliations:** 1 CSIR-Center for Cellular and Molecular Biology, Hyderabad, India

## 

Large proportion of the genome in higher eukaryotes does not code for proteins. This non-coding DNA is emerging as key to the packaging of the genome in form of chromatin, the functional form of genome, that is critical for chromosomal organization and gene regulation. It is clear that the packaging of the genome has regulatory consequences. We, however, do not know what the ‘*packaging code’* of genome is, that allows as many packaging options as the number of cell types in the organism. What is clear is that packaging restricts enhancers/silencers that are capable of functioning over long distances, to interact with only appropriate promoters. Boundary elements that define topologically independent chromatin domains are expected to flank each gene or gene complex that is differentially regulated. Using comparative genomics, large scale mapping of epigenetic modification and genetic approaches we identified a large number of boundary elements by mapping epigenetic chromatin features across the hox complexes of vertebrates and use transgenic approaches to functionally analyze them. We find that the regulatory elements that are involved in the regulation genes by higher order chromatin structure are conserved across species - from flies to mouse. Finally, we use powerful genetic approaches in *Drosophila melanogaster* to explore the epigenetic mechanisms involved in the genomic packaging and regulation of genes. Our findings highlight long-range interactions involved in regulation of genes by means of genomic packaging in cell type specific manner. We propose that accumulation of non-coding DNA, including at least some of the repetitive elements, with the evolution of complexity is then consequence of the regulatory function embedded in this part of genome.

